# The Senolytic Drug Fisetin Attenuates Bone Degeneration in the *Zmpste24*^*−/−*^ Progeria Mouse Model

**DOI:** 10.1155/2023/5572754

**Published:** 2023-02-22

**Authors:** William S. Hambright, Xiaodong Mu, Xueqin Gao, Ping Guo, Yohei Kawakami, John Mitchell, Michael Mullen, Anna-Laura Nelson, Chelsea Bahney, Haruki Nishimura, Justin Hellwinkel, Andrew Eck, Johnny Huard

**Affiliations:** ^1^Steadman Philippon Research Institute, Center for Regenerative Sports Medicine, 181 W. Meadow Dr, Vail, CO, USA; ^2^Department of Orthopaedic Surgery, Kobe University Graduate School of Medicine, 1-1 Rokkodai-cho, Nada-ku, Kobe 657-8501, JP, Japan; ^3^Department of Bioengineering, Colorado State University, 225 Scott Bioengineering Building, Fort Collins, CO 80523, USA; ^4^University of California San Francisco, Orthopaedic Trauma Institute, 1500 Owens Street, San Francisco, CA 94158, USA; ^5^Department of Orthopedic Surgery, Columbia University, Irving Medical Center, New York Presbyterian Hospital, 622 W 168^th^ St, New York, NY, USA; ^6^Department of Orthopaedic Surgery, McGovern Medical School, University of Texas Health Science Center at Houston, 7000 Fannin St, Houston, TX 77030, USA

## Abstract

Aging leads to several geriatric conditions including osteoporosis (OP) and associated frailty syndrome. Treatments for these conditions are limited and none target fundamental drivers of pathology, and thus identifying strategies to delay progressive loss of tissue homeostasis and functional reserve will significantly improve quality of life in elderly individuals. A fundamental property of aging is the accumulation of senescent cells. Senescence is a cell state defined by loss of proliferative capacity, resistance to apoptosis, and the release of a proinflammatory and anti-regenerative senescence-associated secretory phenotype (SASP). The accumulation of senescent cells and SASP factors is thought to significantly contribute to systemic aging. Senolytics—compounds which selectively target and kill senescent cells—have been characterized to target and inhibit anti-apoptotic pathways that are upregulated during senescence, which can elicit apoptosis in senescent cells and relieve SASP production. Senescent cells have been linked to several age-related pathologies including bone density loss and osteoarthritis in mice. Previous studies in murine models of OP have demonstrated that targeting senescent cells pharmacologically with senolytic drugs can reduce symptomology of the disease. Here, we demonstrate the efficacy of senolytic drugs (dasatinib, quercetin, and fisetin) to improve age-associated degeneration in bone using the Zmpste24^−/−^ (Z24^−/−^) progeria murine system for Hutchinson–Gilford progeria syndrome (HGPS). We found that the combination of dasatinib plus quercetin could not significantly mitigate trabecular bone loss although fisetin administration could reduce bone density loss in the accelerated aging Z24^−/−^ model. Furthermore, the overt bone density loss observed in the Z24^−/−^ model reported herein highlights the Z24 model as a translational model to recapitulate alterations in bone density associated with advanced age. Consistent with the “geroscience hypothesis,” these data demonstrate the utility of targeting a fundamental driver of systemic aging (senescent cell accumulation) to alleviate a common condition with age, bone deterioration.

## 1. Introduction

Age-associated degeneration is responsible for a majority of the morbidity, mortality, and healthcare costs worldwide. Aging predisposes people to problems including osteoporosis (OP), osteoarthritis (OA), frailty, and many others that negatively impact people's physical, mental, and emotional health [1]. The CDC estimates that over 55 million people are impacted by OP in the United States, and by the year 2040, it is estimated to increase to over 78 million people. Worldwide it is estimated that there is an osteoporotic fracture every 3 seconds with over 158 million individuals falling into a high risk category for low bone mass-related fracture [2, 3]. Senescent cell accumulation is a fundamental property of aging [4–7] and has been implicated in numerous age-related morbidities [8–10] including OP [11–15]. Cellular senescence is a cellular process distinguished by metabolic inactivity and lack of cell division with distinct phenotypic changes including increased expression of *p16*^*Ink4a*^, significant secretome changes, telomere erosion, and the decondensation of chromatin [16]. Senescent cells release a senescence-associated secretory phenotype (SASP) that is thought to promote aging largely through chronic induction of inflammation. It has been shown in naturally aged mice (24 months) that *p16*^*Ink4a*^ expression is significantly increased in several cell types related to bone biology such as T cells, myeloid lineage cells, osteoprogenitor cells, and even osteocytes [17]. Senescence has been linked to orthopedic decline with aging. Premature cellular senescence has been found to be present during OA pathogenesis [18, 19], and transplantation of senescent cells in knees of mice with OA was found to elicit pain, decreased mobility, and OA-like radiographic and histological signatures [20–22]. Senescent cells have also been linked to OP [23] and frailty [24, 25], and targeting SASP pharmacologically via JAK inhibition has been demonstrated to alleviate bone density loss in aged mice [12]. Thus, senescent cells seem to have a fundamental role in the natural aging process and in age-related orthopedic conditions, including OP and frailty, highlighting senescence as a promising therapeutic target.

Current pharmacological intervention in OP aims to mitigate the incidence of bone fracture in patients either by blocking osteoclast activity with anti-resorptive drugs or by giving osteoanabolic drugs to stimulate bone formation [26]. Long-term use of either drug class remains limited due to serious side effects including atrial fibrillation, thromboembolism, clinical symptoms in the kidneys and gastrointestinal tract, esophageal cancer risk, and potential appearance of osteonecrosis and osteosarcoma [27]. Furthermore, anti-resorptive drugs are often associated with a simultaneous decrease in bone formation which can hinder fracture healing [28]. As such, there remains a significant unmet clinical need for disease-modifying alternatives that preserve bone formation with a better safety profile.

Senolytic drugs have been found to directly target and clear senescent cells globally without affecting healthy non-senescent dividing cells [9, 13]. Senolytic drugs thus offer a potentially novel therapeutic approach for the treatment of OP via targeting a conserved aging property that promotes pathogenesis, senescence, and resulting SASP. Senolytic drugs such as dasatinib (D), quercetin (Q), and fisetin (FIS) have been found to eliminate senescent cells both *in vitro* and *in vivo* [9, 13]. Dasatinib is an FDA-approved treatment for leukemia [29], while quercetin and fisetin are naturally occurring compounds that are tolerable at relatively high doses [30–32]. Importantly, these drugs hit different anti-apoptotic pathways, so a combination would allow for a multi-hit approach to deal with the complexities of the different SASPs produced and anti-apoptotic pathways hijacked by senescent cells. The combination of D/Q was recently demonstrated to significantly prevent cortical and trabecular bone resorption in naturally aging mice but have yet to be studied in progeroid mice [12]. The effects of FIS on bone integrity and regeneration have not been studied, although it was recently shown that FIS could extend health and lifespan in mice [33]. Pharmacological removal of senescent cells with these senolytic compounds has widespread systemic benefits largely through the reduction in trans-acting SASP factors [9, 10, 13, 24, 34, 35]. Another potential benefit to senolytic therapy is potential to achieve therapeutic efficacy with only intermittent dosing as the anti-apoptotic pathway only needs to be briefly disrupted to clear senescent cells, thereby lessening the side-effect burden in patients [10]. Also, drug resistance is less of a concern considering senescent cells cease to divide and thus cannot acquire selection pressure mutations that may allow resistance such as those found in cancers or with infectious species.

The Zmpste24^−/−^ (Z24^−/−^) murine system of Hutchinson–Gilford progeria syndrome (HGPS) mimics age-associated musculoskeletal deficits in an accelerated and predicable system [36]. Z24^−/−^ mice exhibit similar symptoms of accelerated bone density loss and spontaneous fractures similar to HGPS patients which makes the system an excellent practical and functional tool to test the efficacy of novel therapies.

The model exhibits several established hallmarks of aging including DNA instability, epigenetic changes, dysregulated nutrient sensing, cellular senescence, loss of stem cell function and number, and telomere erosion [36–39]. These phenotypes appear within 3-4 months of age, making the Z24^−/−^ model a practical tool for aging research, and we have an established cohort on site. The increase of deregulated lamin A/C processing in these mice leads to the destabilization of chromatin and DNA damage resulting in premature senescence. It has also been demonstrated that reduced lamin A/C can lead to reduced osteogenesis and increased adipogenesis [40–42] in bone and that frail individuals have reduced circulating osteoprogenitors [43] with reduced lamin A/C [44]. Thus, the Z24^−/−^ model might better recapitulate conserved aging-associated cellular and systemic factors promoting OP and provide an innovative tool to enable more rapid testing of pharmacologics to treat age-related bone attrition. Here, we report that FIS administration could mitigate bone density loss in the accelerated aging model. These findings highlight the potential for senotherapies in the setting of OP with aging.

## 2. Methods

### 2.1. Zmpste24^−/−^ (Z24^−/−^) Mice

*Z24* ^*−/−*^ progeria mice were previously generated as described with homozygous deletion of *Zmpste24* [36]. Mice were historically developed on a background of 50% C57BL/6 and 50% 129 SvJae. For these studies, *Zmpste24*^*−/−*^ mice were made via crossing *Zmpste24*^*−/+*^ heterozygotes and using *Zmpste24*^*+/+*^ (WT) control littermates. For histology of the skeleton, 8-week-old mice were sacrificed and skeletons were fixed in 95% ethanol. Alcian blue and Alizarin red staining was performed as described [37]. All animal experiments followed approved procedures according to the UTHealth IACUC standards.

### 2.2. Quantitative Real-Time PCR (qPCR)

10–30 mg whole kidney tissue was homogenized in Trizol for 2 min. Total RNA was extracted using Trizol following manufacturer's instructions, and cDNA was generated using the qScript cDNA synthesis kit (QuantaBio). Gene expression was determined for senescence transcripts via qPCR using the StepOnePlus platform (Applied Biosystems). Relative gene expression was determined using the delta-delta Ct method as described. Murine primers are as follows: p16 (F-GTCGCAGGTTCTTGGTCACT, R-CGAATCTGCACCGTAGTTGA), IL-6 (F-CTTCCATCCAGTTGCCTTCT, R-ACAGGTCTGTTGGGAGTGGT), and TGF-*β* (F-CTGCTGACCCCCACTGATAC, R-CAACCCAGGTCCTTCCTAAA).

### 2.3. Senolytic Drug Treatments In Vitro

MC3T3-E1 murine pre-osteoblasts (calvarial) were purchased from ATCC (CRL-2593). ATDC5 murine chondrocytes were purchased from ECACC (99072806). Passage 4–12 cells were seeded overnight at 10,000 cells/well in 24-well plates and then treated with senolytic drugs for 48 h. Cells were cultured in proliferation media, *α* MEM (Invitrogen, A10490-01), 10% fetal bovine serum (FBS), and 1% penicillin/streptomycin for MC3T3 cells and DMEM/F12 (Ham) (1 : 1) (Invitrogen, 11320-033), 10% fetal bovine serum (FBS), and 1% penicillin/streptomycin for ATDC5 cells. Concentrations of senolytic drugs were consistent for all cell types: dasatinib/quercetin (D/Q, 200 nM/20 *µ*M and 400 nM/20 *µ*M) and fisetin (FIS, 25 *µ*M and 50 *µ*M). Viability was assessed following senolytic drug treatments for all cell types using PrestoBlue Cell Viability Dye Reagent (Invitrogen, A13261) following manufacturer's instructions.

### 2.4. C_12_FDG Senescence Detection Using Flow Cytometry

Following senolytic drug treatment, MC3T3 and ATDC5 cells were washed and then treated with the fluorescent senescence stain C_12_FDG (33 *µ*m, ThermoFisher, Waltham, MA, #D2893) in culture media for 2 hours in 5.0% CO_2_. Cells were then trypsinized, washed, and reconstituted in PBS. C_12_FDG positive cells were detected using a 6HT-2L Guava easyCyte flow cytometer (Luminex, Austin, TX) using PBS only and C_12_FDG unlabeled cells as negative controls.

### 2.5. Senolytic Drug Treatments In Vivo

Adult progeroid Z24^−/−^ mice were administered senolytic drugs via oral gavage (100–150 *µ*L volume) starting at 2.5 to 3.0 months of age. Dasatinib/quercetin (DQ, 5 mg/kg; 50 mg/kg in 10% PEG400/5.0% DMSO) was given as a single dose at 2.5 months or multi-dose at 2.5, 3.0, 3.5, 4.0, 4.5, and 5.0 months. Both single and multi-dose DQ mice were sacrificed at 5 months. Fisetin (FIS, 100 mg/kg in 90% PEG400/10.0% ethanol) treated mice were given a total of 4 weekly doses of drug starting at 3.0 months of age and then sacrificed at 4.0 months of age. Additionally, mice were treated with alvespimycin or 17-dimethylaminoethylamino-17-demethoxygeldanamycin (17-DMAG) 3 times per week (10 mg/kg, 1% DMSO-PBS) starting at 2 months of age and then sacrificed at 4 months.

### 2.6. Histological Evaluation of Cartilage

Knee joints from *Z24*^*−/−*^ animals were decalcified in 10% ethylenediaminetetraacetic acid disodium (EDTA) that contained 1.0% sodium hydroxide for a total of 6 weeks. Samples were then dehydrated and paraffin embedded for sagittal visualization of the knee joint. To investigate articular cartilage, 5 *μ*m sections were cut and stained with Alcian blue (to detect acidic glycosaminoglycans (GAGs) or mucopolysaccharides) and Safranin O (to detect proteoglycan content) following established protocols as described [45, 46]. Images were captured with a Leica DMIRB microscope and Retiga digital camera with Northern Eclipse software (v6.0; Empix Imaging, 200x magnification).

### 2.7. *µ*CT Analysis

For DQ and 17-DMAG treatments, mice were euthanized at 4 months of age (*n* = 3/group) and tibia and spine were collected and fixed in formalin for 2 days. A Viva CT 40 scanner (Scanco Medical, Switzerland) at 15 *µ*m with 70 kVP/112 *µ*A X-ray energy was used for measurements. To analyze trabecular bone, we performed measurements on the 3^rd^ lumbar (L3) vertebral body. Trabecular bone was analyzed with the following: Gauss = 0.8, Sigma = 1, and threshold = 163. Cortical bone was analyzed with the following: 50 slices immediately distal to the proximal tibia growth plate, Gauss = 0.8, Sigma = 1, and threshold = 200. Microarchitectural analysis was performed using a direct (no model) approach as previously described [47, 48]. For FIS treatments, mice were imaged using the eXplore Locus Ultra Pre-Clinical CT Scanner (GE Healthcare, London, ON) through the pre-Clinical CT Core Facility with the acquisition settings of 140 kVp and 22 mA with 16 s rotation/exposure. Non-cardiac gated CT images were also collected for every timepoint. Simple back projections were collected using the 0.154 *µ*m image reconstruction and exported as DICOM images prior to analysis using OsiriX software. Qualitative analysis of the bones was performed in 2D and 3D reconstructions using OsiriX. MicroView imaging software was also used for the bone mineral density measurements.

### 2.8. Statistical Analysis

One-way analysis of variance (ANOVA) followed by Tukey's Honestly Significant Different (HSD) post hoc multiple comparison test was used when analyzing quantitative data (multiple) with GraphPad Prism 7. Two-tailed Student T testing was utilized when comparing two groups, and Wilcoxon rank-sum test was used for non-parametric testing as in *µ*CT and histology scoring which was because of the increased deviation of parameters for these outputs as described previously [47, 48].

## 3. Results

### 3.1. Zmpste24-Deficient Mice Exhibit Musculoskeletal Abnormalities Associated with Senescence

**Figure 1:**
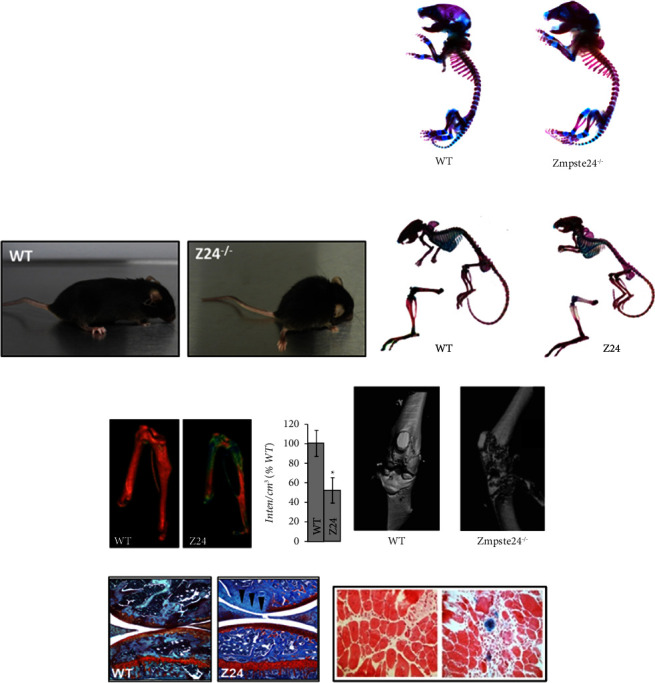
Musculoskeletal abnormalities in *Zmpste24*^*−/−*^ (Z24^−/−^) mice. (a) 4-month-old WT versus Z24^−/−^ mice indicating decreased weight and hunched posture. (b) Representative Alizarin red and Alcian blue staining of 4 mo WT and Z24^−/−^ skeletal preparations. (c) Micro-CT morphometry of hindlimbs indicating significant decrease in trabecular bone density in 4 mo Z24 mice versus age-matched WT (*n* = 6, ^*∗*^*P* ≤ 0.05). (d) Representative radiographic images of Z24^−/−^ knee joint versus WT showing symptoms of OA. (e) Representative Safranin O staining highlighting spontaneous proteoglycan loss in articular cartilage of 4 mo Z24^−/−^ mice (*n* = 3, *P* ≤ 0.05). (f) Representative image of skeletal muscle section demonstrating senescent cell presence in WT versus Z24^−/−^ mice (*n* = 3, ^*∗*^*P* ≤ 0.05).

The Z24^−/−^ progeria model provides an appealing and practical pre-clinical tool to evaluate musculoskeletal aging phenotypes. Z24^−/−^ mice begin to exhibit significant musculoskeletal decline as early as 2 months and have a lifespan of approximately 6 months [36]. Z24^−/−^ mice demonstrate significant weight loss (Figure 1(a)), bone density loss (Figures 1(b)–1(d)), and spontaneous OA-like symptoms including accelerated loss of proteoglycan content (Figure 1(e)) associated with age and senescence (Figure 1(f)). Overall, adult Z24^−/−^ mice exhibit several age-associated musculoskeletal conditions including decreased bone formation which are associated with senescence. Thus, the Z24^−/−^ model provides a practical tool to investigate musculoskeletal pathologies and explore treatment options for not only conditions that occur during natural aging but also patients with HGPS.

### 3.2. Senolytics Effectively Eliminate Senescent Cells in Cultured MC3T3 Osteoblasts and ATDC5 Chondrocytes

**Figure 2:**
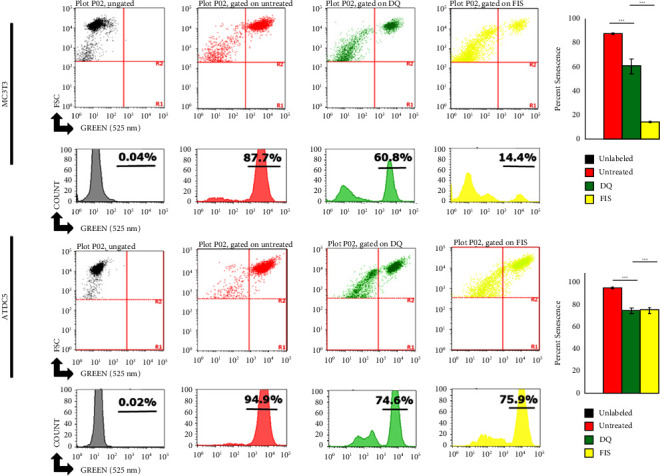
Senolytic drugs reduce senescent cell number in MC3T3 osteoblasts and ATDC5 chondrocytes. Representative flow cytometry plots indicating gating strategy for MC3T3 and ATDC5 cells stained for 1 h with the florescent senescence marker C_12_FDG following treatment with either DQ (200 nM + 20 *μ*M) or FIS (50 *μ*M) for 48 h. Unstained plots represent cells not exposed to C_12_FDG used to exclude debris. Histograms indicating shift in % senescent cell population in cells treated with D + Q or FIS. Quantification reflects multiple experiments from passage (∼8) per cell type with 3 wells per treatment, ^*∗∗∗*^*P* ≤ 0.001.

It is currently unknown which cell types are principally affected by senolytic treatments *in vivo* that may lead to musculoskeletal protection. To assess senolytic treatments directly on cells important for musculoskeletal health (e.g., cartilage and bone cells), we treated murine chondrocyte (ATDC5) and pre-osteoblast (MC3T3) cell lines. For each cell line, cells were grown to passages at 20% O_2_ when senescent cells were found to accumulate (∼passage 10). Confluent cells were treated for 48 h with either DQ or FIS (D/Q, 200 nM/20 *µ*M; FIS, 50 *µ*M). Cells were stained with the senescence marker C_12_FDG, trypsinized, and then analyzed for positive signal using flow cytometry. As expected, both DQ and FIS treatments were able to significantly reduce senescent cell burden in ATDC5 chondrocytes and MC3T3 pre-osteoblasts (Figure 2) demonstrating that both DQ and FIS could reduce senescence in cells important for musculoskeletal regeneration. Proliferation rates and senescence changes with different senolytic doses were previously generated in ATDC5 cells and bone marrow-derived MSCs for the selection of final senolytic doses (Supplemental Figures 1–4).

### 3.3. Senolytic Treatment with DQ or 17-DMAG Is Not Protective against Bone Density Loss in Z24^−/−^ Mice

**Figure 3:**
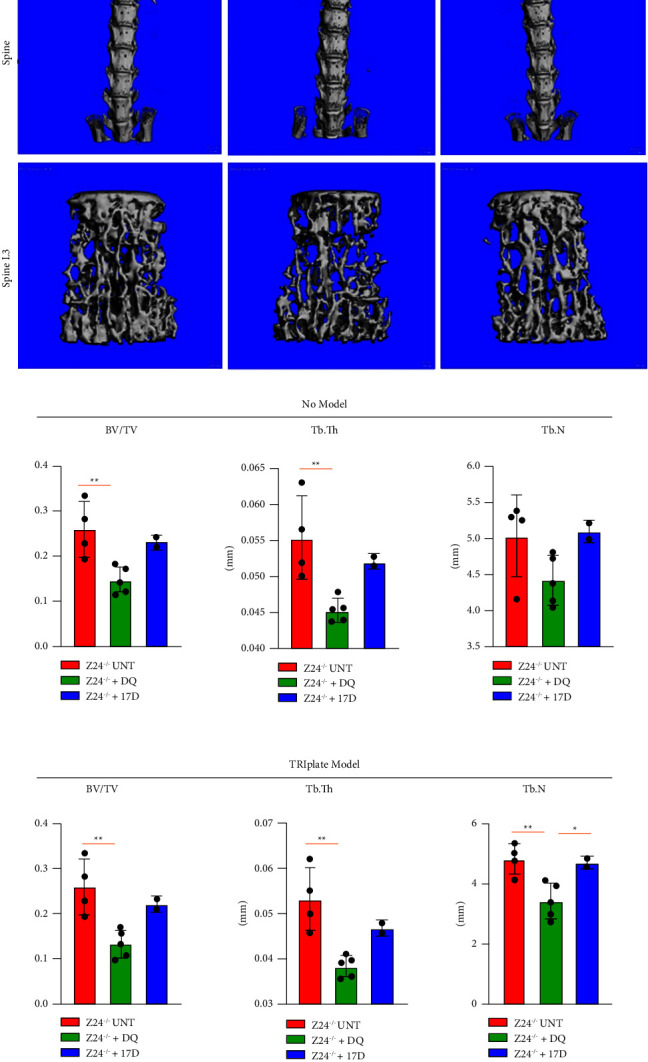
Differential effects of other senolytic drugs on bone loss in Z24^−/−^ mice. (a) Micro-CT-generated bone morphometry of spine from Z24^−/−^ mice treated with DQ or 17-DMAG (17D) starting at 3 months of age. (b) Quantified trabecular number (Tb. N), trabecular thickness (Tb. Th), and bone volume fraction (BV/TV) per senolytic therapy (UNT, *n* = 4; DQ, 5 mg/kg D 50 mg/kg; Q *n* = 5; 17-DMAG, 10 mg/kg, *n* = 2; ^*∗∗*^*P* < 0.01, ^*∗*^*P* < 0.05).

The combination of DQ has previously been shown to mitigate bone density loss in cortical and trabecular bone in naturally aged mice [12]. Considering the positive effects of DQ on cartilage found in Z24^−/−^ mice, we next considered the effects of DQ on bone density loss observed in the model. Bones were dissected from a subset of DQ-treated mice at 5 months of age, a time when significant weight loss and skeletal abnormalities are known to occur, and bone morphometry analysis was performed using *µ*CT. Interestingly, when comparing trabecular bone density of spine, we found no obvious significant protection from bone loss in Z24^−/−^ mice treated with DQ versus age-matched untreated Z24^−/−^ mice (Figure 3(a)). Quantification of trabecular number (Tb. N), trabecular thickness (Tb. Th), and bone volume fraction (BV/TV) using no model or the plate modeling [49, 50] indicated no significant protection from bone loss in the progeroid animals (Figure 3(b)). 17-DMAG (alvespimycin) is another senolytic drug that sensitizes senescent cells to apoptosis through a different inhibitory pathway (HSP90) than DQ [51]. However, bone morphometry analysis for Z24^−/−^ mice treated with 17-DMAG bi-weekly for 10 weeks yielded similar results with limited protection from bone density loss (Figures 3(a) and 3(b)). Interestingly, there was often significantly more bone loss in both DQ- and 17-DMAG-treated Z24^−/−^ mice following the outlined dosing. However, this may have been due to treatment chronicity, dosage, or that pathways targeted by DQ and 17-DMAG may not be principal drivers of senescence in the Z24^−/−^ progeria model. Overall, these data suggest that neither DQ nor 17-DMAG has the potential to mitigate bone density loss in the Z24^−/−^ HGPS model.

### 3.4. Fisetin Treatment Prevents Bone Density Loss in Z24^−/−^ Mice

**Figure 4:**
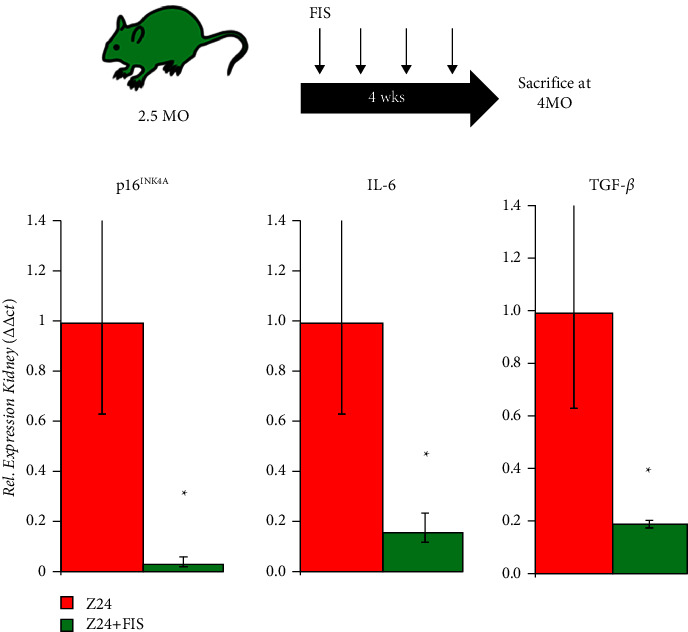
Fisetin reduces senescence-associated transcripts. (a) Weekly dosing strategy for FIS treatments. (b) qPCR data for senescence marker p16INK4A and proinflammatory SASP factors IL-6 and TGF-*β*. Quantification based on ΔΔCt method for relative gene expression (*n* = 3, ^*∗*^*P* ≤ 0.05).

**Figure 5:**
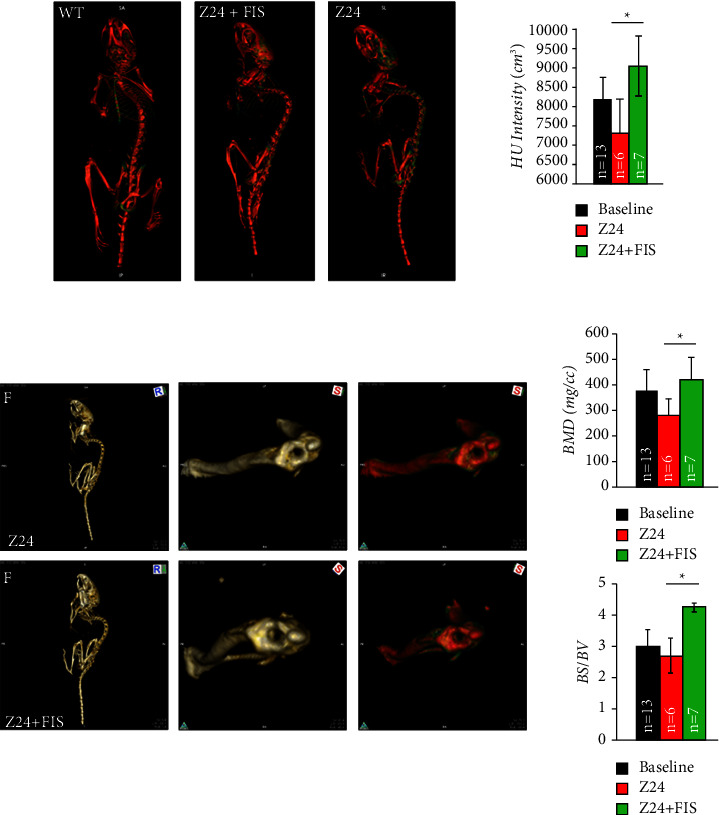
Acute fisetin treatment attenuates bone loss in Z24 mice. (a) Micro-CT images of whole skeleton for 4 mo Z24^−/−^ mice with and without fisetin (FIS) versus age-matched WT. (b) Micro-CT-generated bone morphometry of tibiofemoral joint (knee) of Z24 mice with or without 1-month fisetin treatment. Quantification of micro-CT imaging for Hounsfield unit (HU) intensity, bone mineral density (BMD), and specific bone surface (BS/BV). Quantification based on nontreated Z24^−/−^ control mice (*n* = 7, ^*∗*^*P* ≤ 0.05).

While no significant protective effects in bone were observed using DQ, we next considered the use of the phytonutrient fisetin. Fisetin was chosen for several reasons. One, it was recently demonstrated to increase the healthspan and lifespan of progeroid mice [33], two, it targets separate anti-apoptotic pathways upregulated in senescent cells (SIRT1 [52], BCL-2/BCL-X_L_ [35, 53], and HIF-1*α* [54]), and three, it is a much less toxic alternative to D and 17-DMAG which may have explained the lack of protection in the frail Z24^−/−^ model. Z24^−/−^ mice were treated with FIS weekly for 4 weeks (100 mg/kg) starting at 3 months of age and then sacrificed at 4 months of age (Figure 4(a)). 4 months was chosen as the sacrifice time (versus 5 months) given the more advanced pathology that occurred at 5 months, a time when therapies might have negligible effects as seen in DQ-treated animals. To first identify if senolytic treatment could reduce senescent indices systemically, transcript levels of senescence markers were measured in kidney tissue, an organ known to be sensitive and indicative of senotherapies. It was found that Z24^−/−^ mice treated with FIS had reduced transcript levels of the senescence marker p16^INK4a^ and SASP factors IL-6 and TGF-*β* in the kidney (Figure 4(b)). Importantly, it was found that weekly dosing of FIS for weeks starting at 3 months of age could significantly attenuate bone density loss in Z24^−/−^ mice as evidenced by *µ*CT analysis (Figure 5). Z24^−/−^ mice treated with FIS had significantly higher scores for Hounsfield unit (HU) intensity, bone mineral density (BMD), and specific bone surface (BS/BV) versus untreated Z24^−/−^ mice. Thus, unlike DQ, senolytic therapy with FIS protected against age-related bone density loss in the accelerated aging Z24^−/−^ background suggesting the importance of senescence to bone loss during aging.

## 4. Discussion

In elderly individuals, fractures due to osteoporosis (OP) and functional deficit represent a significant healthcare burden. Thus, novel treatment strategies for age-related bone loss are desperately needed, especially those that may be disease modifying as opposed to the treatment of symptoms. Cellular senescence is considered to be a significant contributor to age-related dysfunction in musculoskeletal tissues including bone and articular cartilage [11–15]. Cellular senescence in OP is known to be associated with increased SASP, decreased proliferative activity of bone resident cells, decreased bone density, diminished proteoglycan content and degeneration of hyaline cartilage, and increased senescence-associated transcripts in bone and joint tissues. Here, we demonstrate that the Zmpste24^−/−^ (Z24^−/−^) murine model of HGPS can be used as a predictable accelerated aging system to model age-related decline in bone density.

The Z24^−/−^ model exhibits multiple established hallmarks of aging and senescence, including musculoskeletal deterioration [36–39] that appears within 3-4 months of age, making the Z24^−/−^ model a practical tool for aging-related musculoskeletal decline. Here, bone density of Z24^−/−^ mice was found to be significantly reduced compared to WT mice. Combining these findings with increased senescence associated transcripts *p16*^*INK4a*^ and p21Cip1 in tissues and isolated primary stem cells, we propose Z24^−/−^ mice to serve as a rapid and predictable model to study various therapies targeting senescence including senolytics. Senolytic drugs that selectively remove senescent cells pharmacologically offer a very appealing and novel therapeutic modality for the treatment of pathologies of age. Dasatinib (D), a pan-tyrosine kinase inhibitor, is an FDA-approved treatment for leukemia [29] while quercetin (Q) targets several anti-apoptotic nodes PI3K [9, 10], HIF-1*α* [10, 54], and MDM2/p53/p21/serpine (PAI-1 and 2) [9, 10]. Fisetin (FIS) has been shown to modulate signaling pathways disrupted during senescence such as SIRT1 [52], BCL-2/BCL-X_L_ [35, 53], HIF-1*α* [54], MDM2/p53/p21/serpine (PAI-1 and 2) [35, 55], and AKT [35, 56] leading to clearance of senescent cells, decreases in reactive oxygen species (ROS) production, and reduction in SASP driven inflammation *in vitro* and *in vivo*. Both FIS and Q are also phytonutrients with significantly better safety profiles than chemotherapeutic drugs like 17-DMAG or D [30–32]. Besides off-target side effects, senolytic agents exhibit significant variance in efficacy depending on cell or tissue type [13]. This is likely because not all senescent cells are the same. Some express different SASP factors, have different senescent markers, or hijack different mechanisms to avoid apoptosis [10, 34]. Thus, safer senolytic alternatives, or combination senolytic therapies, are likely essential in older patients to safely and systemically remove senescent cells from multiple tissues [10, 12, 13]. In addition, because senolytic drugs lack the need to be constantly bioavailable to have the desired anti-senescent effect, treatments can be intermittent to reduce potential side effects and minimize patient interactions [9, 10].

While anti-resorptive therapies for OP exist, their association with concomitant reduction in bone formation limits means that treatment is typically stopped following fracture to facilitate bone healing [28]. Our data showing increased BMD following senotherapy in the Z24^−/−^ mice suggest that this a potentially safer treatment of low bone mass. Previous reports have shown that DQ can ameliorate bone density loss in naturally aged mice [12]. Interestingly, our findings indicated that DQ had minimal effects on mitigating bone loss in Z24^−/−^ mice as seen by *µ*CT of the tibia and spine. However, acute FIS treatment was found to significantly increase bone density of the whole skeleton and protect against bone density loss in Z24^−/−^ mice. These results may in part be due to the ability of FIS to better target senescent osteoblast cells as seen by DQ and FIS treatments in MC3T3-E1 cells. Here, it was found that FIS effectively eliminates senescent osteoblasts more readily than DQ as seen by C_12_FDG staining. This leads to a proposed combinatorial approach for treating age-related musculoskeletal decline, postulating that combinatorial senolytic therapies may allow for a multi-hit approach in targeting bone loss. Senolytic drugs may thus offer a novel approach to target OP or other age-related musculoskeletal conditions such as OA. In OA for example, cartilage degeneration is the irreversible culprit for pain, inflammation, and functional decline [57]. Intervention to regenerate articular cartilage by restoring healthy chondrocyte-mediated extracellular matrix (ECM) production is the holy grail for potential OA therapies. Stem cell-based therapies to regenerate articular cartilage as well as mechanical, biological, or chemical scaffolds to restore ECM-like function have not been able to reverse OA or restore mechanical properties of the joint [57]. Thus, senolytics, which are known to restore and enhance endogenous progenitor cell function, may translate nicely in this context.

## 5. Conclusions

In summation, we have shown that senolytic agents can reduce age and senescence-associated musculoskeletal pathology in an accelerated aging model. Cellular senescence is a fundamental regulator of healthspan and lifespan. By targeting such fundamental aging processes, this study highlights senolytic drugs as a safe pharmacological alternative that could transform geriatric medicine by simultaneously preventing several age-related diseases, syndromes, and deficits including bone and cartilage degeneration. In addition, current therapies for OP are lacking in that they often are associated with side effects from chronic use and do not target etiological drivers of disease. Several senotherapeutic drugs have optimal safety profiles. Natural compounds such as fisetin and quercetin are over-the-counter dietary supplements with minimal side effects with evidence from this study and others highlighting their efficacy to mitigate age-related musculoskeletal decline.

## Data Availability

Raw data collected not reported herein will be provided upon request.

## Supplementary Materials

Supplementary Materials Supplemental Figure 1: proliferation effects of fisetin versus vehicle controls at different doses. Supplemental Figure 2: dose effects of fisetin on freshly harvested bone marrow concentrate (BMC). Supplemental Figure 3: dose response in ATDC5 chondrocytes treated with varying concentrations of D + Q. Supplemental Figure 4: senolytic drugs reduce viability in cultured osteoblasts and chondrocytes.
