# Supplementary material for: The Senolytic Drug Fisetin Attenuates Bone Degeneration in the Zmpste24−/− Progeria Mouse Model
Source: J Osteoporos. 2023 Feb 22;2023:5572754. doi: 10.1155/2023/5572754 (PMC9977556; doi:10.1155/2023/5572754)
Supplement: Supplementary Materials — Supplemental Figure 1: proliferation effects of fisetin versus vehicle controls at different doses. Supplemental Figure 2: dose effects of fisetin on freshly harvested bone marrow concentrate (BMC). Supplemental Figure 3: dose response in ATDC5 chondrocytes treated with varying concentrations of D + Q. Supplemental Figure 4: senolytic drugs reduce viability in cultured osteoblasts and chondrocytes. [file 5572754.f1.docx]

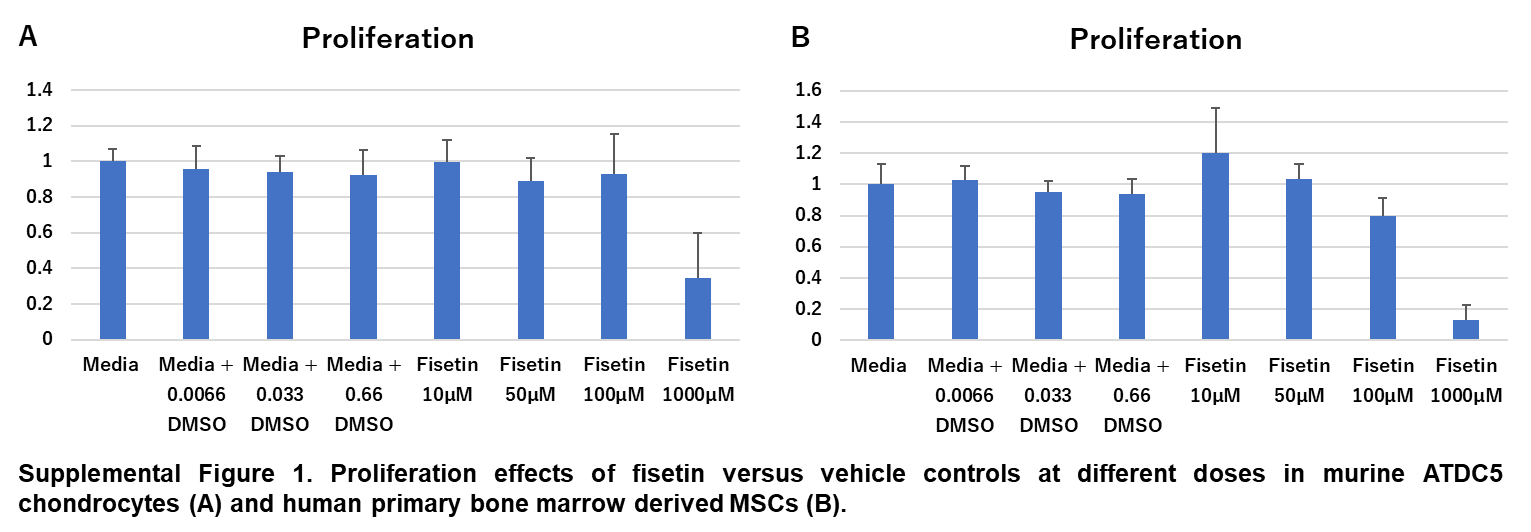


**Supplemental Figure 1. Proliferation effects of fisetin versus vehicle controls at different doses in murine ATDC5 chondrocytes (A) and human primary bone marrow derived MSCs (B).**

**
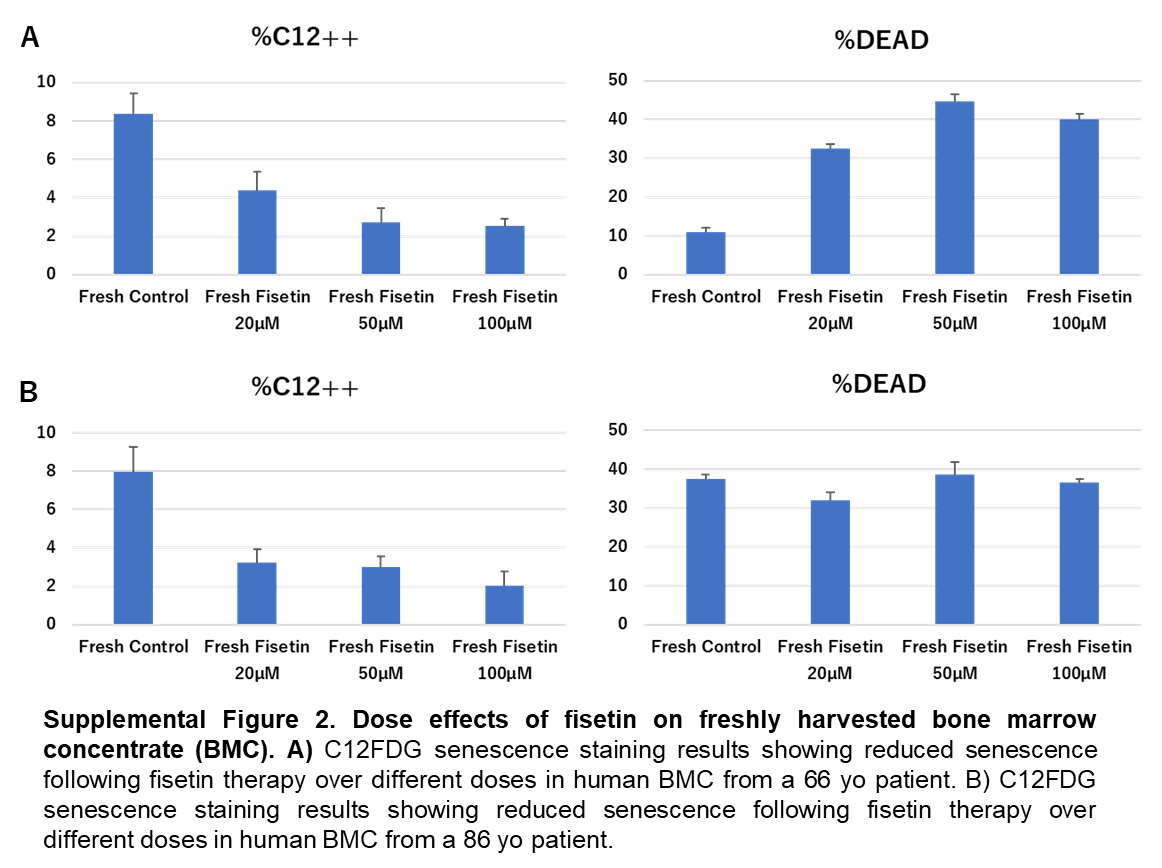
**

**Supplemental Figure 2. Dose effects of fisetin on freshly harvested bone marrow concentrate (BMC).** A) C12FDG senescence staining results showing reduced senescence following fisetin therapy over different doses in human BMC from a 66 yo patient. B) C12FDG senescence staining results showing reduced senescence following fisetin therapy over different doses in human BMC from an 86 yo patient.

**
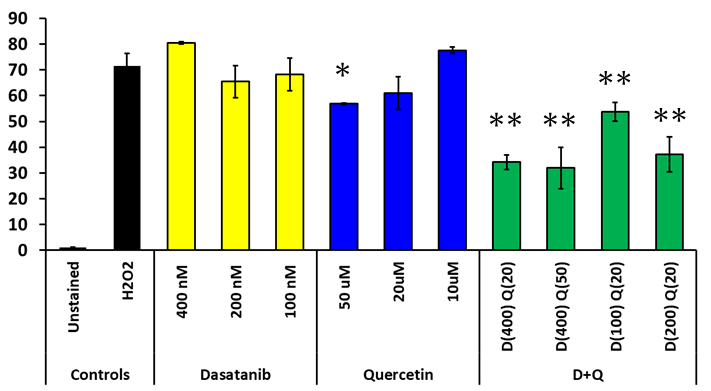
**

**Supplemental Figure 3. Dose response in ATDC5 chondrocytes treated with varying concentrations of D+Q.** Quantified % senescence cells for ATDC5 cells (passage 4) treated with dasatinib (D) or quercetin (Q) alone and in combination for 48 hrs indicating optimal senescence reduction with co-treatment (D+Q). % senescence was detected using flow cytometry following 1 hr incubation with the fluorescent senescence marker C_12_FDG (525 nm). Quantification reflects multiple experiments from different passages per cell type with 3 wells per treatment, **P* < 0.05, ***P* < 0.01.

**
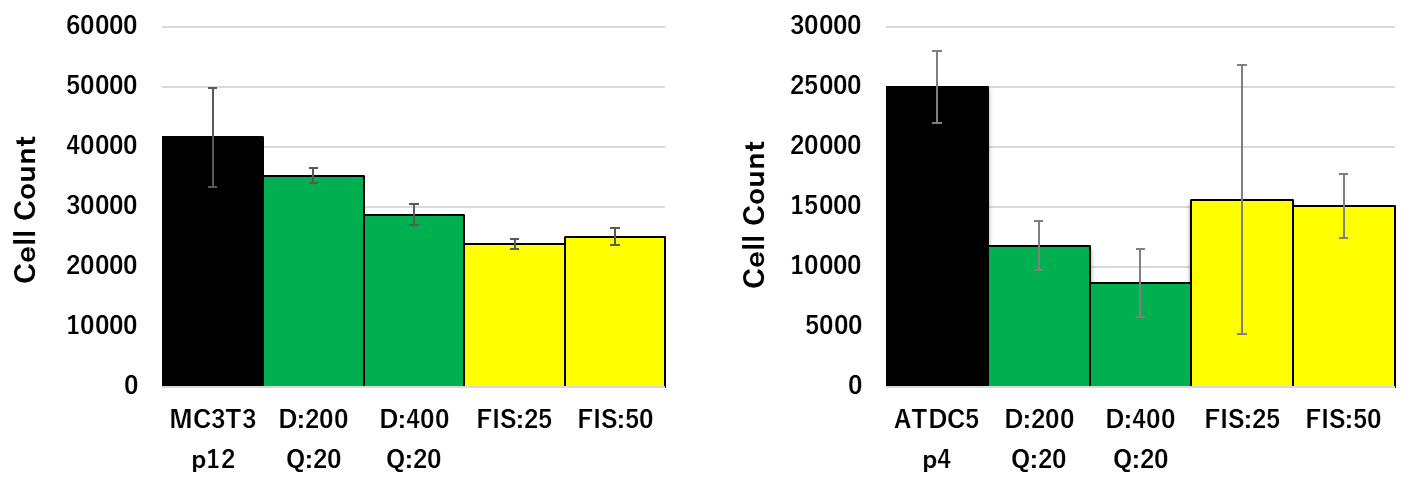
**

**Supplemental Figure 4. Senolytic Drugs Reduce Viability in Cultured Osteoblasts and Chondrocytes.** Effects of senolytic drug treatment on cell viability at different doses using the PrestoBlue assay. MC3T3 cells (passage 12) and ATDC5 cells (passage 4) treated with different doses of dasatinib (D), quercetin (Q) or fisetin (FIS) for 48 hrs. Quantification reflects multiple experiments from different passages per cell type with 3 wells per treatment.
